# Timely and Efficient Infant Diagnosis Is Required to End AIDS in Children

**DOI:** 10.1002/jia2.70113

**Published:** 2026-06-19

**Authors:** Lara Vojnov, Ilesh V. Jani

**Affiliations:** ^1^ Global Health Impact Group Atlanta Georgia USA; ^2^ Instituto Nacional de Saúde Marracuene Mozambique

**Keywords:** birth testing, case‐finding, confirmatory testing, diagnostic integration, end AIDS, infant diagnosis, innovations, paediatric HIV, point‐of‐care

## Abstract

**Introduction:**

Though considerable effort and investment has significantly increased the proportion of pregnant women living with HIV accessing lifesaving antiretroviral therapy and lowered the prevalence of vertical HIV transmission, more than 70,000 children die each year due to AIDS‐related deaths, and nearly half of the 1.4 million children living with HIV are not receiving antiretroviral treatment (ART). Improving case‐finding and identification of children acquiring HIV and accelerating linkage to ART will be fundamental to end AIDS in children.

**Discussion:**

A variety of existing infant and child case‐finding strategies could be tailored by countries to complement testing within vertical HIV transmission programmes, including establishing the HIV exposure status of sick infants and children attending malnutrition and inpatient wards and children accessing vaccines, as well as out‐of‐facility strategies such as community and family‐based testing. Because the risk of HIV acquisition is persistent throughout pregnancy, delivery and breastfeeding, repeated testing throughout the exposure period and especially after cessation of breastfeeding will be critical in capturing HIV acquisitions as early as possible. Immediate or rapid linkage to ART is now possible through using same‐day point‐of‐care testing technologies.

**Conclusions:**

As countries aim to end AIDS in children, adapted and nuanced strategies for case‐finding, test timing and the technologies used should be leveraged to meet the needs of each setting and country. Several countries have shown us that it is possible to end AIDS; however, it requires political will, strategic thinking, funding, prioritization and creative innovations.

## 1 Introduction

Prevention of vertical transmission of HIV has improved steadily over the last 25 years, especially due to universal access to antiretroviral treatment (ART) in high‐burden countries. Globally, 84% of pregnant women living with HIV are on ART, while 93% of pregnant women living with HIV are on ART in eastern and southern Africa [1]—the majority of which access optimized regimens that include integrase inhibitors such as dolutegravir. Still, in 2024, an estimated 120,000 children acquired HIV through vertical transmission, and 75,000 children died due to AIDS‐related causes. Although these figures represent a substantial drop since 2010, HIV acquisition remains an important contributor to infant and child mortality [1].

Though access to ART for infants and children has increased in the last decade, it has not expanded at the same rate as that for adults [1, 2]. Currently, nearly half of the 1.4 million children living with HIV globally are not receiving life‐saving ART. Without treatment, 50% of children who acquired HIV perinatally die by their second birthday, with mortality peaking at 2−3 months of age [3, 4].

Early diagnosis is a critical step in the identification and treatment of children living with HIV. Several technological and systems innovations have increased access to and the quality of HIV diagnosis in children. Nevertheless, gaps remain in providing HIV diagnosis services that are efficient, timely and accessible to all in both low‐ and high‐prevalence settings.

Recent and ongoing international donor funding cuts have severely disrupted health services [5, 6, 7], with children living with HIV being significantly affected. Between the end of 2024 and the first 3 months of 2025, new paediatric ART initiations fell in several countries, as did the number of children on ART, while the number of children lost to follow‐up increased [8]. Consequently, countries are optimizing, reprogramming and reprioritizing activities to maintain public health gains and keep long‐term ambitions on track.

Despite persistent challenges and gaps, and as countries advance closer towards elimination of paediatric AIDS, it will be essential to focus on key interventions to identify new HIV acquisitions in infants and children and to ensure ongoing and uninterrupted care in those already diagnosed, limiting associated morbidity and mortality.

## 2 Discussion

The single most critical intervention to prevent vertical HIV transmission is early and ongoing ART initiation in women of childbearing age. High viral loads due to incident acquisitions in pregnancy or breastfeeding, as well as in mothers not on ART, contribute to over 90% of new HIV acquisitions among children [1]. While testing pregnant women during the first antenatal care visit should continue to be a mainstay, HIV retesting during late antenatal care as well as at delivery or 6 weeks postpartum have been found to be the most effective testing intervention to reduce vertical transmission in high‐prevalence settings [9]. Once identified, providing consistent and reliable access to optimized ART for women living with HIV, ideally before conception or early in pregnancy, provides the best opportunity to prevent vertical transmission [10]. Once daily dolutegravir‐based ART regimens are now widely available, highly effective, well tolerated and with a high barrier to drug resistance, increasing viral suppression rates and decreasing the risk of vertical transmission if taken as prescribed [11]. Improving and prioritizing infant diagnosis should be coupled with ART delivery to women living with HIV to quickly identify infants and children living with HIV and in need of subsequent treatment and care.

### 2.1 Where to Test: Case‐Finding

Traditionally, case‐finding is based on a nucleic acid‐based test at 4–8 weeks of age for infants known to be exposed to HIV and enrolled in prevention of vertical transmission programmes [11]. However, as vertical transmission rates decline due to the success of maternal ART and prevention programmes, additional case‐finding strategies should be considered to achieve the ambitious goals set forth by the Global Alliance to End AIDS in Children by 2030 [11, 12, 13].

For the optimal prognosis, infants and children at risk of HIV acquisition would be identified and tested early, and those living with HIV would be identified while healthy for quick linkage to ART. Capturing the parent(s) and child's HIV status at any healthcare touchpoint, such as the Essential Programme on Immunization and well‐child visits, will support improved linkage to necessary follow‐up services such as HIV testing [11, 12].

Expanding infant and child case‐finding strategies during fiscal constraints will require cost‐efficient and integrated approaches. For example, in countries with high HIV burdens and low or modest malnutrition rates, establishing the HIV exposure status of all infants and children attending malnutrition wards or inpatient settings could significantly improve identification of children living with HIV [11, 12]. However, doing so would rely on integrated healthcare systems that ensure linkage of infants across essential services. Furthermore, less traditional, out‐of‐facility case‐finding approaches, such as community testing, family testing and testing of children of new cases of people living with HIV, could improve convenience for the family, while sensitizing them to the needs of their child(ren), and improve case‐finding [11]. Understanding HIV prevalence rates across a variety of community‐ and facility‐based settings will guide countries interested to expand testing beyond prevention of vertical transmission programmes.

Vertical disease programmes are likely to face significant challenges moving forward due to international donor funding cuts and shifting priorities. This may also constitute an opportunity for countries to develop and provide better, more holistic, universal care through system‐wide provision of services. Integrating HIV and other health services has been shown to improve health outcomes and the sustainability of the HIV response [14].

### 2.2 When to Test: Test Timing

Infants are continuously susceptible to HIV acquisition during pregnancy, delivery and breastfeeding. The likelihood of vertical transmission during each of these stages depends on antenatal care attendance, maternal access to ART, access to viral load testing, maternal ward attendance for delivery, infant prophylaxis provision, breastfeeding practices, and retention in continued HIV care and treatment throughout the duration of exposure. However, most infant HIV diagnostic testing occurs at the 4–8 weeks of age time point, per WHO recommendations and global targets and indicators [11, 15]. Identifying HIV acquisition as early to the event as possible can increase the child's prognosis [16, 17] and requires several tests throughout the period of exposure.

Testing exposed neonates in high‐prevalence settings using nucleic acid assays identifies more than half of the HIV acquisitions detected at 4–8 weeks of age [18, 19]. Birth testing is accurate and allows ART initiation in the first weeks of life, thus preventing the early mortality peak [11, 20]. Yet, few countries in sub‐Saharan Africa have implemented routine birth testing. The main constraints include financial resources to add another testing point and the challenges in administering ART to neonates [11]. While promising recent developments in neonatal ART might be an incentive, current resource constraints may continue to pose a formidable barrier to scale‐up birth testing.

For children acquiring HIV during breastfeeding, testing at the 4–8 weeks of age time point is too early, as it can only diagnose HIV acquired during pregnancy or delivery. Because a substantial proportion of children either acquire HIV during breastfeeding or were missed for testing at the traditional time point, it is essential for countries to expand testing until the end of cessation of breastfeeding. This could include multiple tests during the exposure period or at least one after the cessation of breastfeeding. To support widespread uptake of later infant diagnosis, national and global programmes should include an indicator for end‐of‐exposure testing and prioritize retention in care for HIV exposed children. Ending AIDS in children may not be achievable without a comprehensive HIV testing strategy encompassing the whole period between birth and the end of breastfeeding for all exposed infants. Beyond clinical morbidity and mortality, improved support for both mother and infant is critical given the often‐complicated treatment and care journeys—stigma, discrimination, trauma while navigating different treatment practices over the past 5–10 years and in some cases, on a per pregnancy case.

### 2.3 How to Test: Available Testing Technologies

HIV diagnosis for infants and children under the age of 18 months has typically been conducted using dried blood spot samples taken at the point‐of‐contact and shipped to centralized laboratories for nucleic acid testing [21]. Results are then transmitted, either by paper return or electronically, to the healthcare facility and eventually to the child's caregiver. This testing modality revolutionized infant HIV care more than a decade ago by providing a diagnostic means to accessing ART in primary healthcare facilities. Many countries continue to rely on centralized testing as the backbone of their infant diagnosis programme. As maternal antibodies wane in young children, serological‐based rapid diagnostic tests can be used from 18 months of age onwards to diagnose HIV acquisition.

Though turnaround times of testing within the laboratory have been shortened considerably in recent years, sample transportation networks and result delivery mechanisms continue to plague efficient clinical decision‐making for these vulnerable children. The introduction of molecular point‐of‐care infant HIV testing now allows providers to test and initiate treatment on the same day for children under 18 months of age, a paradigm previously only available for older children and adults [11, 22]. Children living with HIV were nine times more likely to initiate ART within 60 days when tested using a same‐day result return point‐of‐care test compared to the traditional centralized testing model [22]. However, widespread use of these technologies has been hampered primarily by high device and test prices. Fortunately, considerable product development and investment is ongoing in new point‐of‐care and near point‐of‐care molecular technologies for other diseases (e.g. tuberculosis) that could incorporate an infant HIV assay—diagnostic integration or sharing of platforms has been found to be a cost‐saving approach [11, 23, 24].

Most molecular technologies currently available for infant diagnosis are platforms that can be shared across programmes, including tuberculosis, cervical cancer, hepatitis and HIV viral load [24]. Sharing devices and associated systems and services, such as sample transportation, quality management and so on, across diseases and programmes is cost‐saving and creates efficiencies within national public health programmes [11, 23, 24]. Furthermore, it provides programmes with the opportunity to access a larger footprint of devices.

In the future, disposable molecular technologies may significantly narrow the gaps of incomplete identification and lower ART initiation rates for children in resource‐limited settings. Suitable technologies were developed during the COVID‐19 pandemic [25], however, at relatively high costs and for high‐income settings. Ongoing work between suppliers, product development partners and funders, national governments and global stakeholders will be critical to bring these innovative technologies to market for the most vulnerable.

### 2.4 Additional Considerations as Vertical Transmission Decreases

As vertical transmission rates begin to decrease to less than 5% in high‐burden countries, testing options and algorithms need to be adjusted to ensure the highest performance and accuracy. Initiating lifelong ART for a child falsely identified as having acquired HIV has considerable and lasting impacts. Conversely, missing a case of acquired HIV also has devastating consequences. All HIV virological tests available and listed by WHO prequalification fulfil WHO recommendations to have a sensitivity of at least 95% and specificity of 98% or higher [26]. The specificities as listed in the WHO prequalification public reports for each listed assay are 100%, with lower confidence interval bounds of 97% or higher. However, the positive predictive value of a test, that is a child who tests positive actually has acquired HIV, becomes more critical as vertical transmission rates decrease. For example, the positive predictive value of a test with 98% specificity in a setting with a prevalence rate of 2% will be 50.3%—meaning that half of the positive tests will be true positives and the other half false positives [27]. Implementing a two‐test algorithm would increase the positive predictive value in that hypothetical setting to 97.9%, significantly reducing the number of false positives identified and potentially put on lifelong ART [27]. Countries can increase the positive predictive value in the face of lowering vertical transmission rates by ensuring all positive tests are repeated with any remaining sample and/or confirmatory tested with a fresh sample [11, 27]. If challenges persist in executing these strategies, an indeterminate range can be applied to reduce the number of false positives [11, 28, 29].

Finally, the benefits of improved coverage and access to infant diagnosis can only be realized by ensuring consistent and reliable linkage to care and treatment. Technology‐based solutions, health system integration and community support interventions can all support enhanced and rapid linkage to care. For example, as mentioned earlier, point‐of‐care technologies to support immediate same‐day treatment initiation, while mobile health systems can improve communication and faster return of results [30]. Incorporating peer mentor mothers in the case management of mothers and their children has been shown to increase retention in care [31]. Additionally, health system integration through integrating HIV testing and treatment services of infants and children into existing maternal and child health services leverages high attendance rates [14], task‐sharing expands the available workforce capacity [32], and optimizing sample transportation for faster sample and result delivery can each support improved testing service quality. Furthermore, acknowledging the considerable lifelong challenges of mothers and their children living with HIV is a start to providing more comprehensive, supportive and stigma‐free care for both groups.

## 3 Conclusions

**FIGURE 1:**
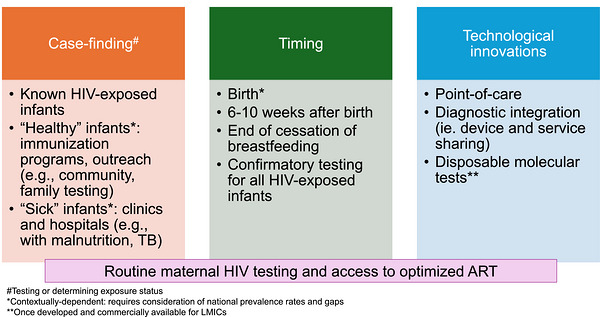
Tools and innovations to improve identification of infants and children living with HIV in integrated health systems. Abbreviations: ART, antiretroviral therapy; TB, tubercolosis.

Several interventions to close the ongoing gaps and challenges in ensuring access to HIV testing in children are available for country adaptation (Figure 1). First and foremost, ensuring all women are treated with optimized ART for their own health will also protect more infants from acquiring HIV. Reaching those marginalized or failing to attend regular ante‐ and post‐natal health visits through community outreach will be essential to this effort. For infants and children at risk of acquiring HIV, designing the most effective, context‐specific case‐finding strategies that complement traditional testing within vertical transmission programmes will expand access to and coverage of infant HIV diagnosis; however, no one‐size approach will fit all settings. Developing more integrated programmes and services will require a paradigm shift, but ultimately, ensure all healthcare needs are met for infants and children. This starts with understanding the HIV status of all infants and children in high‐burden countries, especially those who are sick. In most settings, there is a need for testing infants and children known to be exposed to HIV multiple times from birth to cessation of breastfeeding. Finally, incorporating same‐day point‐of‐care infant HIV testing should be prioritized for all national programmes—it is an ethical right for infants and children to be afforded the same standard provided to adults. Under the current fiscal constraints, as national reprogramming and integration of services becomes a reality, children must continue to be our highest priority.

## Author Contributions

LV and IVJ: Conceived of the commentary, drafted, revised and approved of the manuscript.

## Conflicts of Interest

The authors declare no conflicts of interest.

## Data Availability

Data sharing not applicable to this article as no datasets were generated or analysed during the current study.
